# Surgical options in retrosternal oesophageal reconstruction

**DOI:** 10.1007/s00423-024-03433-6

**Published:** 2024-08-03

**Authors:** Lars Kollmann, Sven Flemming, Johan Friso Lock, Armin Wiegering, Christoph-Thomas Germer, Florian Seyfried

**Affiliations:** 1 Department of General, Visceral, Transplantation, Vascular, and Paediatric Surgery, University Hospital Wuerzburg, Wuerzburg, Germany; 2 Comprehensive Cancer Centre, Wuerzburg, Germany; 3 Head of Surgery for Upper Gastrointestinal Tract and Metabolic Surgery, Department of General, Visceral, Transplantation, Vascular and Paediatric Surgery, Centre of Operative Medicine (ZOM), University Hospital of Wuerzburg, Wuerzburg, Germany

**Keywords:** Colonic interposition, Discontinuity, Esophageal resection

## Abstract

**Background:**

Retrosternal oesophageal reconstructions with collar anastomoses can become necessary when the stomach is either unavailable for oesophageal replacement, or orthotopic reconstruction is deemed impractical. Our aim was to analyse our results regarding technical approaches and outcomes.

**Materials and methods:**

All patients undergoing primary and secondary oesophageal retrosternal reconstructions with collar anastomoses at our centre (2019–2023) were retrospectively analysed and individual surgical reconstruction options were presented.

**Results:**

Overall, twelve patients received primary (*n* = 5; 42.7%) or secondary (*n* = 7; 58.3%) reconstructions; ten with colonic interposition and two with gastric pull-up. Male/female ratio was 4:8; median age 66 years (30–87). Charlson-Comorbidity-Score (CCS) was 5 (1–7); 8/12 patients (67%) had ASA-classification score ≥ 3. We observed no conduit necrosis, but one patient (8.3%) with a leakage of the oesophago-colonostomy which was successfully treated by endoscopic vacuum therapy. Four patients (33.3%) acquired nosocomial pneumonia. Additional drainages for pleural fluid collections were necessary in three patients (25%). Overall comprehensive-complication-index (CCI) was 26.2 (0–44.9). Length-of-stay (LOS) was 22 days median (15-40). There was no 90-days mortality. Overall, CCI during the follow-up (FU) period at median 26 months (16–50) was 33.7 (0–100). 10 out of 12 patients were on sufficient oral nutrition at 12 months FU.

**Conclusion:**

Primary and secondary oesophageal retrosternal reconstructions encompass diverse entities and typically requires tailored decision-making. These procedures, though rare, are feasible with acceptable complication rates and positive functional outcomes when performed in experienced hands.

**Supplementary Information:**

The online version contains supplementary material available at 10.1007/s00423-024-03433-6.

## Introduction

The orthotopic gastric pull-up with intrathoracic anastomosis has become the gold standard surgical reconstruction technique for esophageal-cardia resections [1]. This operation is increasingly offered either as a hybrid or entirely minimally invasive, mainly robotic-assisted, procedure, thereby reducing perioperative morbidity and mortality in certified centres [2]. The advantage of the minimal invasive access, coupled with the necessity of only a single anastomosis post-gastric tube formation, is noteworthy.

However, situations arise where the stomach is either unavailable, or orthotopic reconstruction, such as post-salvage esophagectomy, is deemed impractical [3]. These scenarios encompass diverse entities and typically necessitate tailored decision-making concerning the optimal approach for each individual patient [4].

Various reserve techniques with collar reconstruction are available, yet they are infrequently performed even in specialised centres [3]. This retrospective case series meticulously reviews all such consecutive cases from our tertiary referral and oesophageal cancer centre accredited by the German Cancer Society over the past four years, examining individual baseline scenarios, indications, technical approaches, and perioperative and functional outcomes.

## Methods

### Patient cohort

All patients who underwent collar oesophageal anastomosis (colon interposition or gastric pull-up) between 2019 and 2023 were prospectively recorded and retrospectively evaluated. This study was approved by the the local ethics committee. Preoperative assessment included a nutritional status and, where necessary, additional enteral or parenteral nutrition. A gastric or jejunal feeding catheter for enteral alimentation was in place in all patients after salvage esophagectomy without primary reconstruction. Preoperative colonoscopy and CT scan of the abdomen and chest was mandated for all patients (not more than two years prior), followed by bowel preparation immediately before surgery, administered through the enteral feeding catheter in cases of discontinuity resection.

Details about individual patient history, indication and technical considerations and surgical details are provided in Table 1.

**Table 1:** Individual case description

No.	Age	Initial disease	Type of initial index Surgery/intervention	Indication for reconstruction	Type of Reconstruction	Individual description
1	69	EGJ Carcinoma	Ivor Lewis with subsequent anastomotic stenosis along with delayed gastric emptyping	Pseudoachalasia	Colon interposition	Chronic (8 years) therapy-refractory stenosis of the oesophagogastrostomy combined with delayed gastric emptying, secondary retrosternal Colon interposition (Roux-en-Y).
2	66	Boerhaave syndrome	Salvage oesophagectomy	Esophageal discontinuity	Gastric pull-up	Emergency esophagostomy in septic multi-organ failure due to Boerhaave syndrome, secondary reconstruction with gastric pull-up.
3	70	Incarcerated upside- down stomach	Merendino with subsequent leakage of oesophagojejunostomy	Esophageal discontinuity	Colon interposition	Leakage after Merendino procedure due to incarcerated upside-down stomach years after sleeve gastrectomy, salvage oesophagectomy, secondary colon interposition onto the merendino-limb.
4	62	Long segment EGJ Carcinoma	oncological D2 gastrectomy with partial oesophagectomy	Stomach not available for reconstruction	Colon interposition	Oncological D2 Gastrectomy along with thoracal partial oesophagectomy for a long-distance longitudinally spread cardiac carcinoma after neoadjuvant treatment. Followed by reconstruction via colon interposition with distal Roux-en-Y anastomosis.
5	68	Long segment EGJ Carcinoma	oncological D2 gastrectomy with partial oesophagectomy	Stomach not available for reconstruction	Colon interposition	Oncological D2 Gastrectomy along with thoracal partial oesophagectomy for a long-distance longitudinally spread cardiac carcinoma after neoadjuvant treatment. Followed by reconstruction via colon interposition with distal Roux-en-Y anastomosis.
6	74	Hiatal hernia	Fundoplication with early recurrence and incarceration of the stomach along with esophageal perforation	Esophageal discontinuity	Colon interposition	Fundoplication with early recurrence and partial incarceration of the stomach along with esophageal perforation alio loco, salvage oesophagectomy and partial gastric resection, secondary colon interposition.
7	62	Esophageal squamous carcinoma	Hybrid Ivor-Lewis with subsequent eosophago-bronchial fistula	Esophageal discontinuity	Colon interposition	Hybrid Ivor-Lewis after neoadjuvant treated esophageal squamous carcinoma with subsequent oesophago-bronchial fistula, salvage conduit resection and treatment of bronchial fistula, secondary colon interposition onto Roux-en-Y-limb.
8	69	Achalasia	Several Myotomies and hiatal revisions	End-stage-achalasia with sigmoidal transformation	Colon interposition	Elective esophagectomy and colon interposition in end-stage-achalasie with mega-esophagus and poor QoL.
9	61	Esophageal SCC	Hybrid Ivor-Lewis with subsequent eosophago-bronchial fistula	Esophageal discontinuity	Colon interposition	Hybrid Ivor-Lewis after neoadjuvant treated esophageal squamous carcinoma with subsequent eosophago-bronchial fistula alio loco, salvage conduit resection and treatment of bronchial fistula, secondary colon interposition onto Roux-en-Y-limb
10	87	Choledocholithiasis	Iatrogenic esophageal perforation during ERCP	Esophageal discontinuity	Gastric pull-up	Iatrogenic perforation during ERCP for choledocholithiasis, salvage oesophagectomy alio loco. Secondary retrosternal gastric pull-up.
11	30	Achalasia	Several Myotomies and hiatal revisions, partial esophagectomy and gastric pull-up	Therapy refractory severe erosive Reflux disease, sigmoidal transformation of the gastric conduit	Colon interposition	End-stage achalasia; Several Myotomies and hiatal revisions, partial esophagectomy and gastric pull-up with therapy refractory severe erosive Reflux disease along with sigmoidal transformation of the gastric conduit, secondary colon interposition onto Roux-en-Y limb.
12	45	GORD	Fundoplication with multiple hiatal revisions, Merendino with subsequent stenosis and stent perforation into the Merendino limb	Esophageal discontinuity	Colon interposition	Initial Fundoplication with multiple revisions alio loco. Merendino procedure with subsequent stenosis and stent perforation into the Merendino limb Salvage oesophagectomy, Retrosternal colon interposition onto Meredino limb.

Legend: EGJ: esophago-gastral-junction; SCC: squamous cell carcinoma; ERCP: endoscopic retrograde cholangio pancreaticography; GERD: gastro oesophageal reflux disease

### Surgical technique of retrosternal colonic interposition

In all cases, a long left colonic graft was used including the transverse as well as the left colon, the arterial supply origin from the left vessels, and branched in an iso-peristaltic way as previously described [5].

All colon interpositions were performed through a midline laparotomy along with a left cervicotomy for the proximal anastomosis. If a concomitant esophagectomy/resection of the gastric conduit was necessary, it was performed through a right open thoracotomy as previously described [5].

The colon was completely mobilized from the retroperitoneum, starting at the caecum to the sigmoid colon. Then, the arterial vascularisation was identified followed by the occlusion of the right colic artery, the middle colic artery as well as the collateral arcades at both extremities with atraumatic vascular clamps. This manoeuvre has been carried out for at least 10 min to check for sufficient arterial blood supply from the left colonic artery origin from the inferior mesenteric artery as previously reported [6]. During the same time, it was also ensured that there was no venous congestion.

The length of the transplant needed was measured. Then, the proximal and distal end of the graft along with the arcade were transected. The arteria and vena colica media along with the mesocolon were also divided.

A retrosternal position was used as route of reconstruction. The tunnel was created bluntly. A resection of the left part of the manubrium and the clavicula was not performed. The freed colonic transplant, pedicled on the left colonic vessels, was then carefully pulled up and shortened at the oral end to ensure a strait conduit.

**Fig. 1:**
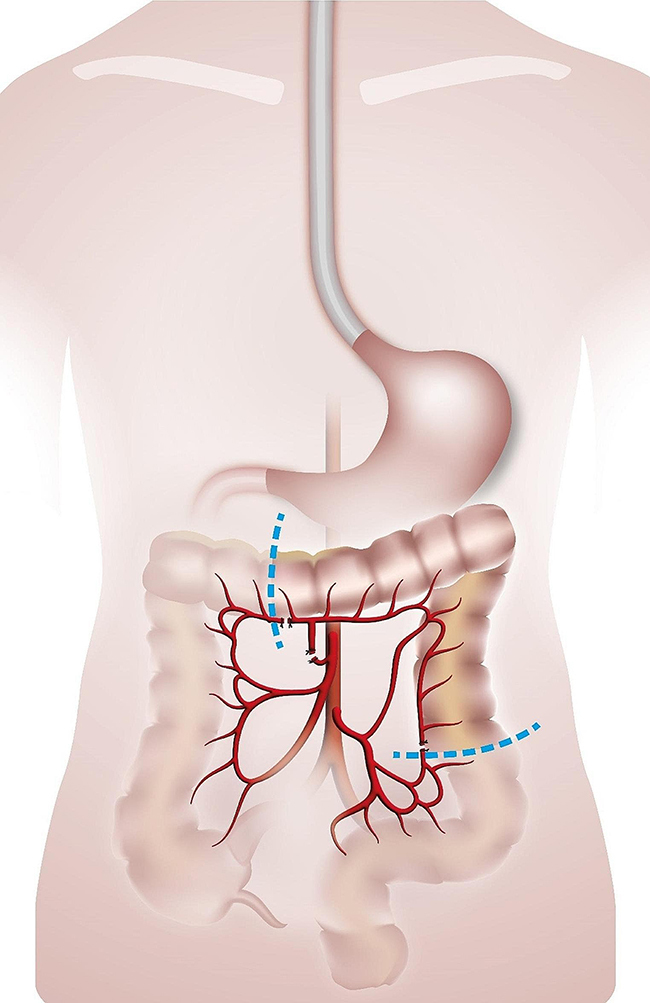
Preparation of a long left colonic graft. Legend: Blue lines represent the proximal and distal end of the long left colonic graft. Medial colonic artery is transected at its origin. The inferior mesenteric artery is preserved and arterial arcade along the proximal and distal extremities is clipped

**Fig. 2:**
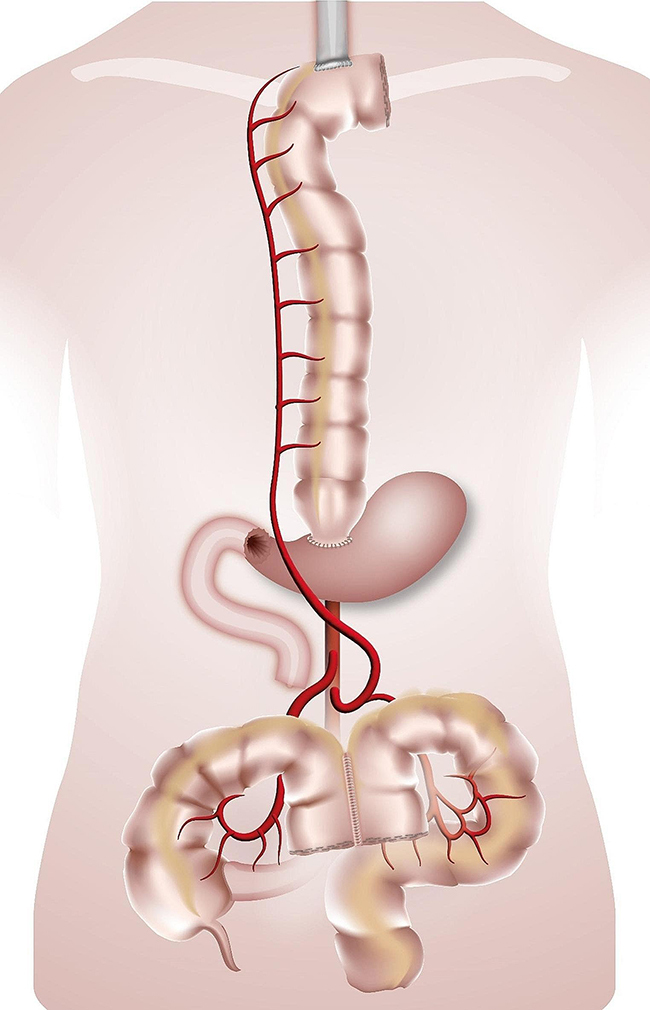
Colonic interposition onto the stomach. Legend: The route for the colonic graft is retrosternal. The graft is anastomized isoperistaltically to the collar oesophagus (termino-lateral) and to the antrum of the stomach (termino-lateral)

**Fig. 3:**
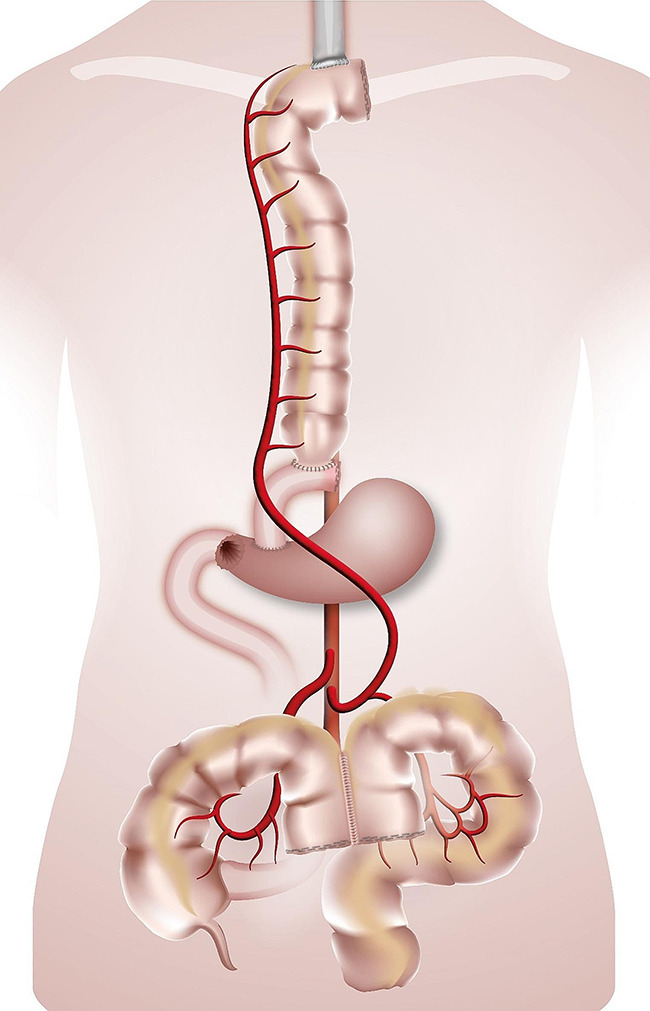
Colonic interposition onto the Merendino limb. The route for the colonic graft is retrosternal. The graft is anastomized isoperistaltically to the collar oesophagus (termino-lateral) and to the exsistinng Merendino limb (termino-lateral). Perfusion by interior mesenteric artery. The transverso-descendostomy is stapled in a latero-lateral fashion

**Fig. 4:**
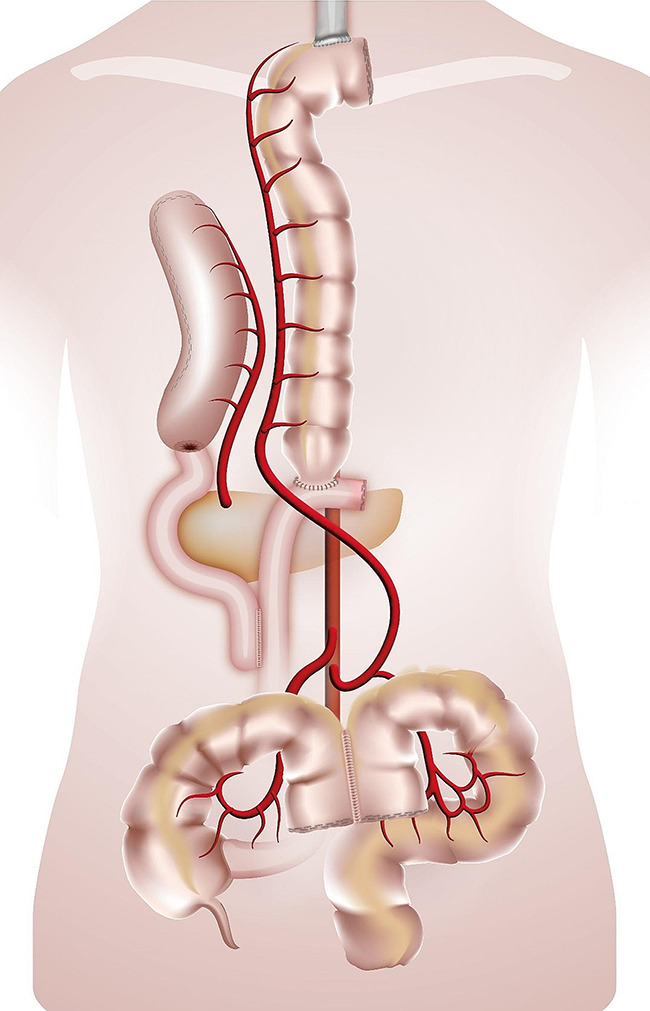
Colonic interposition onto Roux- limb after failed gastric pull-up. The route for the colonic graft is retrosternal. The graft is anastomized isoperistaltically to the collar oesophagus (termino-lateral) and to a Roux-limb (termino-lateral). Perfusion by interior mesenteric artery. The transverso-descendostomy is stapled in a latero-lateral fashion. The exsisting gastric conduit remains in part in situ

The esophago-colonic anastomosis was created hand-sewn in Gambee technique in an end-to side technique fashion. The colon and the anastomosis were then gently retracted into the chest and thus the oesophagus was straightened. The colo-gastric anastomosis was performed hand-sewn or stapled (28 mm Circular Stapler, Covidien) in an end-to-side fashion, the colo-colic one was stapled with a linear stapling device in a side-to-side fashion. When a gastrectomy was performed/or if the stomach has already been used, the colo-gastric anastomosis was replaced with a colo-jejunostomy and a jejuno-jejunal anastomosis (Roux-en-Y loop). In case of a failed Merendino procedure the Merendino-limb was used for the distal anastomosis of the colonic graft. (Figures 1, 2, 3 and 4). A simultaneous cholecystectomy was carried out in all patients if applicable.

### Surgical technique of retrosternal gastric pull-up

During the retrosternal gastric pull-up with cervical anastomosis, it was ensured that the gastro-omental vascular arcade remained intact and that a sufficient Kocher manoeuvre for extensive mobilisation of the duodenum and conduit was performed. Gastric tube formation started below the level of the incisura angularis. The width of gastric tube measured about 4 cm. During mobilization no-touch technique was applied [7].

**Fig. 5:**
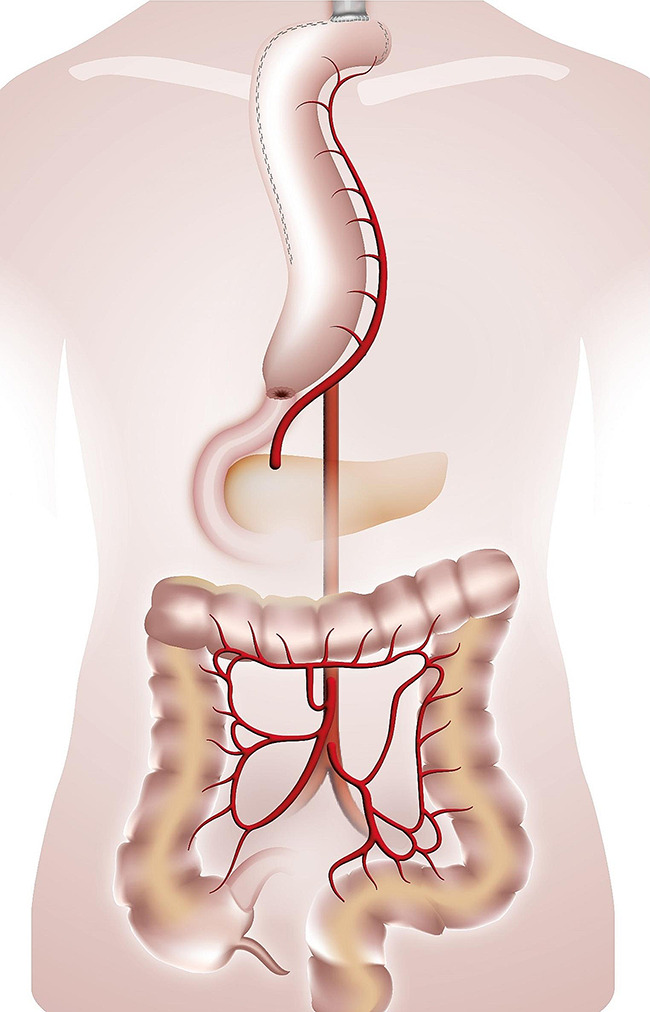
Collar retrosternal gastric pull-up. The gastric pull-up is anastomized termino-lateral to the collar oesophagus. Perfusion by right gastro-omental artery

Spectral imaging methods were not employed; however, adequate arterial inflow was locally verified post-gastric elevation during anastomosis construction. Additional venous (“superdrainage”) or arterial (“supercharged”) microsurgical anastomoses were not utilised. A simultaneous gallbladder removal was also carried out in all patients if applicable. Anatomy is described in Fig. 5.

### Statistical analysis

All statistical analyses were performed using IBM SPSS Statistics 29 (International Business Machines Corporation, Armonk, NY) and Excel 2021 (Microsoft, Redmond, USA). Descriptive data is reported as means with standard deviations, unless otherwise stated. Comparisons between the analysed cohorts were performed using chi-square test, Fisher’s exact test, Mann–Whitney U-test or a one-way analysis of variance in accordance with data scale and distribution. The level of statistical significance was 0.05 (two-sided).

## Results

### Patient cohort

Overall, twelve patients were treated with primary or secondary oesophageal retrosternal reconstruction with collar anastomosis for either oesophageal discontinuity situations (*n* = 7; 58.3%) or primary cancer surgeries where gastric pull-ups were not possible (*n* = 2; 16.7%) and/or poor functional conditions after previous surgeries (*n* = 3; 25%). Male/female ratio was 4:8 (33% vs. 67%). Median age was 66 years (30–87), body-mass-index (BMI) was 22.4 kg/m² (16.7–33.9), Charlson-Comorbidity-Score (CCS) was 5 [1–7] and 8/12 patients (67%) had ASA-classification score ≥ 3. Six patients had cardiac comorbidities (50%), three had COPD (25%), five had a malignant disease (41.7%) of which 2 had squamous cell carcinomas and 3 adenocarcinomas of the oesophagus. One patient was under therapeutic anticoagulation (8.3%) and 4 (33%) had platelet aggregation inhibition. The details are presented in Table 2.

**Table 2:** Patient characteristics

Patients’ characteristics	Number (%) (*n* = 12)
Sex ratio, No. (M: F)	4:8 (33/67)
Age, median (range), years	66 (30–87)
BMI, median (range), kg/m^2^	22.4 (16–34)
Charlson comorbidity score, mean (SD)	5 [1–7]
ASA classification ≥ III	8 (66.6)
Cardiac disease	6 (50.0)
COPD	3 (25.0)
Anticoagulation	1 (8.33)
Platelet aggregation inhibition	4 (33.3)
Indication for retrosternal reconstruction with collar anastomosis:	
- Esophageal discontinuity	**7 (58.3)**
o Complications after functional upper gastrointestinal surgery	2 (16.7)
o Tracheo- or bronchoesophageal fistula	2 (16.7)
o Boerhaave syndrome	1 (8.33)
o Iatrogenic esophageal perforation	1 (8.33)
- Primary oncological resection	**2 (16.7)**
- Functional Disease	**3 (25.0)**
o End-stage Achalasia	2 (16.7)
o Pseudoachalasia after Ivor Lewis	1 (8.33)
History of gastric/oesophageal cancer:	5 (41.7)
- Primary oncological resection with simultaneous reconstruction	2 (16.7)
- Secondary reconstruction for complications after Ivor Lewis	3 (25.0)
Type of previous surgeries:	
- Minimally invasive	1 (8.33)
- Open	9 (75.0)
Index surgery in house	7 (58.3)
Weight loss within 12 weeks prior surgery (in kg)	0 (0–23)
Nutritional deficiencies prior surgery	0 (0)
Nutritional status at primary or secondary retrosternal oesophageal reconstruction:	
- Oral (alone)	4 (33.3)
- Enteral (alone) (feeding tube)	6 (50.0)
- Additional i.v. necessary	2 (16.7)

Legend: M: male; F: female; BMI: body-mass-index; kg: kilogram; SD: standard deviation; ASA: American Society of Anesthesology; COPD: chronic-obstructive-pulmonary-disease; i.v.: intravenous

The number of previous surgeries in our cohort were three (0–15) in median per patient. The approach of previous surgeries was open in 9/12 (75%) patients and one case (8.3%) of minimally-invasive-surgery (MIC). Detailed information of indication and histories are presented in Table 1.

### Nutritional status

Median weight-loss from initial appointment until surgery was zero (0–23 kg). In 4/12 (33%) patients sufficient oral food intake was possible. Six (50%) were fed via a jejunal feeding tube. Two patients received intravenous nutrition. Patients were routinely screened for nutritional deficiencies and treated accordingly. At the time of surgery, none of our patients was in a catabolic state or diagnosed with nutritional deficiencies.

### Operative parameters

Ten (83.3%) patients received long left colonic interposition and two (16.7%) gastric pull-up. In five cases (41.7%) a resection of the oesophagus and/or stomach was performed. The other seven cases received secondary reconstruction alone (58.3%). All esophageal anastomoses were performed hand-sewn in termino-lateral condition. Two colo-gastrostomies (16.7%) and eight colo-jejunostomies (66.7%) were made. In both gastric pull-up esophago-gastrostomies were sutured. The duration of surgery was 341 min (201–721). The gastric pull-ups had significantly shorter duration of surgery (210 vs. 365 min; *p* < 0.001). We had no intraoperative complications in this series. All patients were routinely treated on Intensive-care-unit (ICU) in the postoperative course. Length-of-ICU-stay was 2 days [1–15] in median.

### Postoperative outcome

Complications (measured in the Clavien-Dindo-Classification (CDC) > grade II within 90 days) occurred in six patients (50%). Three patients (25%) had pleural fluid collection which made pleural drainage under local anaesthesia necessary (grade IIIa). One patient (8.3%) suffered from a deep surgical site infection of the thoracotomy (IIIb) which was surgically treated, one patient (8.3%) suffered from multiple infectious complications (IVb) but also developed a leakage of the oesophagocolonostomy which was successfully treated by endoluminal vacuum therapy. Overall, four patients (33.3%) acquired a pneumonia (grade II) which was successfully treated with antibiotics of which one patient was observed on intermediate-care (IMC) ward (grade IVa).

No conduit necrosis or stenosis of the anastomoses were observed. Overall comprehensive-complication-index (CCI) was 26.2 (0-44.9). Length-of-stay (LOS) was 22 days median (15–40). There was no mortality within 90 days. Details are shown in Table 3.

**Table 3:** Operative outcome

Patients’ characteristics	Number (%) (*n* = 12)
Procedure:	
- Colon interposition	10 (83.3)
- Gastric pull-up	2 (16.7)
Resection with primary reconstruction	5 (41.7)
Secondary reconstruction alone	7 (58.3)
Surgical technique (anastomosis)	
- Oesophago-colo/gastrostomy (Hand-sewn termino-lateral)	12 (100)
- Colo-gastrostomy (hand-sewn or stapled termino-lateral)	2 (16.7)
- Colo-jejunostomy (stapled termino-lateral)	8 (66.7)
- Colo-colostomy (stapled isoperistaltic latero-lateral)*	10 (100)*
Duration of surgery (median, range)	341 (201–721)
Intraoperative complications	0 (0)
Reoperation (for surgical site infection)	2 (16.7)
Reintervention**	3 (25.0)
Maximum Level of care (Intensive Care Unit)	12 (100)
Length of ICU stay (days)	2 [1–15]
Postoperative complications (%)	
- Conduit necrosis	0 (0)
- Leakage	1 (8.33)
- Stenosis	0 (0)
- Surgical site infection	2 (16.7)
- Pleural effusion**	3 (25.0)
- Pneumonia	4 (33.3)
Postoperative complications (CDC > grade II) within 90 days	
- CDC IIIa	3 (25)
- CDC IIIb	1 (8.3)
- CDC IVa	1 (8.3)
- CDC IVb	1 (8.3)
Comprehensive Complication Index, ( median, range)	26.2 (0-44.9)
Length of Stay (median, range)	22 (15–40)
Nutritional status at discharge	
- Fully oral nutrition	9 (75.0)
- Additional feeding tube	2 (16.7)
- intravenous	1 (8.33)
Mortality (intrahospital or 90 days)	0 (0)

*: 10/10 cases with colon interposition

**: all reinterventions were for pleural effusions treated with thoracic drain

Legend: ICU: intensive care unit; CDC: Clavien-Dindo-Classification

### Follow-up

The mean interval of follow-up was 26 months (16–50). The main symptoms in FU were unrelated to the surgical technique of reconstruction. Two patients (16.7%) suffered from adhesion ileus and needed surgical treatment. Further two patients (16.7%) needed additional surgeries for tumour recurrences in the follow-up (FU) interval. The patient who developed a late onset leakage developed several high-grade complications during FU and died due to cachexia. One patient developed a stenosis and received repeated endoscopic dilatation (*n* = 3) with full recovery. Overall CCI during FU was 33.7 (0-100). One patient died due to cancer relapse during 12 months FU. 10 out of 10 patients were on sufficient oral nutrition at 12 months FU. Details are shown in Table 4.

**Table 4:** Follow-up-outcome

Patients’ characteristics	Number (%) (*n* = 12)
Interval last FU (months) (mean, range)	26 (16–50)
Major complications during follow-up	
- Ileus	2 (16.7)
- Stenosis*	1 (8.33)
- Hernia	1 (8.33)
- Non related to surgery**	2 (16.7)
- Leakage	1 (8.33)
Weight loss during follow-up (in kg)	0 [1–6]
Additional surgery (%)	5 (41.3)****
-related to CI anastomoses	1 (8.3)***
Additional intervention	1*(8.3)
CCI in FU	33.6 (0-100)
Oral nutrition at 12 months	10 (83.3)

* endoscopic dilatation of esophago-colostomy

** 2 surgeries for metachronous metastases

*** late leakage; multiple revisions, death in follow-up > 90 days < 12months

**** 2 surgeries for metachronous metastases ; 1x multiple revisional thoracotomies due to pleural fistula after initial perforation prior to interposition (leading cause for initial discontinuity); 1x leakage with multiple revisions; 1 incisional hernia surgery

Legend: FU: follow-up; kg: kilogram; CI: colo-intenstinal; CCI: comprehensive-complication-index

### Patient satisfaction

All patients were assessed pre-, and postoperatively regarding their quality of life and satisfaction with the SF-36 form. All patients reported relevantly impaired quality of life (QoL) with the either discontinuity situation or the functional difficulties. Baseline and follow-up results for QoL (all categories of the SF-36) are presented in Table 5.

**Table 5:** Quality of life

Patients’ characteristics	Preoperative (*n* = 12)	Follow-up (*n* = 10)*
Interval last FU (months) (median, range)	0 (0)	26 (16–50)
SF 36 questionnaire		
- Physical functioning	25 (0-100)	50 (0-100)
- Role limitations physical	0 (0–80)	0 (0–75)
- Role limitations emotional	0 (0–70)	33.3 (0-100)
- Energy/Fatigue	10 (0–60)	40 (0–80)
- Emotional wellbeing	40 (0–50)	60 (36–84)
- Social functioning	10 (0–50)	50 (20-87.5)
- Pain	37.5 (0–80)	67.5 (30–100)
- General health	15 (0–80)	35 (15–80)
- Health change	30 (0–50)	55 (25–80)
Oral food intake better than before? (%)	0 (0)	7 (70)
Oral food intake sufficient? (%)	4 (33.3)	10 (100)
Physical condition better than before surgery? (%)	-	6 (60)

*One oncological patient died during FU and one patient refused the evaluation

FU: follow-up; SF 36: short form 36 questionnaire

In supplement Fig. 1a-i the individual changes after retrosternal esophageal reconstruction compared to baseline are shown. After retrosternal esophageal reconstruction oral food intake was sufficient in all and improved in 70% of patients during further follow up.

## Discussion

Our findings corroborate that the necessity for oesophageal reserve reconstructions with cervical anastomosis encompasses a broad spectrum of entities, predominantly involving highly individual and rare circumstances [8, 9]. The most frequent indication was secondary reconstruction after esophageal salvage-resection in benign and malignant conditions. These patients usually have a considerable impaired quality of life and, therefore, often express a strong desire for therapy, notwithstanding the substantial risk of complications associated with complex secondary esophageal reconstructive surgery [10].

However, our results also demonstrate that with meticulous indication, preparation, and surgical execution, good perioperative and functional outcomes can be achieved, albeit such operations are infrequent even in specialized centres [3].

A population-based study from the United Kingdom revealed that the frequency of the procedure did not influence the perioperative outcomes [9]. However, due to the rarity of these procedures, relatively low threshold values were set for low-volume (< 5 procedures) and high-volume (> 10 procedures) centres. The literature reports widely heterogeneous perioperative outcomes, with anastomotic insufficiencies ranging from 3 to 46%, and mortality rates between 0 and 16.7% [11]. In our cohort at a tertiary referral centre, we were able to confirm good perioperative outcomes reported by some centres. The 90-day mortality and conduit ischemia rates were nil, with an anastomotic leakage rate of 8.3%.

Concerning procedure specific details for colon interposition, we are convinced, that meticulous verification of adequate perfusion via the feeding vessel and intact marginal arcade – especially with an intact marginal artery of Drummond - is crucial, as anatomical irregularities are frequently reported in the literature [5]. After complete mobilisation of the entire colon from the retroperitoneum and marking the proximal and distal sites of the interposition, the corresponding marginal arcade and the middle colic artery were temporarily occluded with atraumatic clamps for at least 10 min as previously described [5]. During this manoeuvre, sufficient conduit perfusion was demonstrated in all cases. Selective preoperative angiography was not performed, which some authors recommend in specific situations [12].

Similarly, in retrosternal gastric pull-ups with collar anastomosis, attention was paid to an intact gastro-omental arcade and a sufficient Kocher manoeuvre for extensive mobilisation of the duodenum and conduit. Neither additional venous (“superdrainage”) nor arterial (“supercharged”) microsurgical anastomoses were performed [13, 14].

Our patient cohort predominantly comprised individuals for secondary oesophageal reconstruction after salvage esophagectomy for non-oncological reasons since the primary treatment for anastomotic leaks after Ivor-Lewis surgery at our centre is endoluminal vacuum therapy (EVT), achieving success rates exceeding 98% [15]. Thus, secondary reconstructions due to anastomotic complications after Ivor-Lewis operations were rare, affecting only one of our oncological patients (0.7%) during the entire study period.

The proportion operated patients with oncological history was 41.7% (*n* = 5), with two patients having simultaneous oncological resection, one with therapy refractory pseudoachalasia after Ivor Lewis [16], and two who had undergone esophageal salvage resection for tracheoesophageal fistula (one referred after external primary surgery). Current available literature reports similar proportions of oncological patients undergoing secondary reconstruction [17]. Older reports had higher proportions of oncological cases which may explain poorer outcomes as previously reported [5]. It is noteworthy that the sole patient with an anastomotic leak and a complicated course in our cohort had undergone prior oncological surgery and had multiple risk factors (e.g., neoadjuvant radio-chemotherapy, broncho-oesophageal fistula, asthenia).

Our experience aligns with previous reports highlighting the functional superiority of the colon over the stomach as a substitute for the oesophagus, particularly in younger patients with benign conditions like end-stage achalasia [5]. This was exemplified by a case involving a young female patient with chronic, therapy-refractory severe reflux disease post-gastric pull-up with intrathoracic esophago-gastrostomy, who underwent successful conversion to colonic interposition, yielding a favourable functional outcome.

As a result, gastric pull-up with collar anastomosis was performed exclusively in older patients with a significant risk profile. The advantage of this reconstruction is that potential leaks of the esophago-gastrostomy can usually be treated locally and heal without consequences [15].

In our cohort one leakage of the esophgo-colonostomy occurred which was treated with subsequent EVT treatment (three cycles) without any septic events. This was well tolerated as the anastomosis was at the level of the upper thoracic aperture about seven centimetre distal to the pharynx. If EVT would not have been tolerated the cervical wound would have been opened.

The potential benefits of resecting the sternoclavicular joint in cervical anastomosis were not assessed [11]. However, none of our patients reported swallowing difficulties at the level of the proximal esophagus.

We observed a wide variation in patients’ quality of life prior and after retrocolic esophageal reconstruction. This could be explained by different baseline situations regarding general health, age, the underlying disease, swallowing function and nutritional status. For instance, patients with end-stage Achalasia and patients with secondary reconstructions experienced certain improvements in their quality of life after retrosternal reconstructions [10]. Of note, their SF-36v2 values were comparable to age-matched healthy controls [10]. In contrast, patients needing retrocolic reconstructions with initial good swallowing function and quality of life experienced a certain decrease compared to their previous situation [10]. However, sufficient oral food intake was possible in all patients during follow up.

This study has several limitations due to its design. It is a retrospective single centre case series of 12 patients including vastly different indications and preconditions for primary or secondary esophageal retrosternal reconstructions with cervical anastomoses. Thus, this is more a retrospective descriptive report rather than a comparative study that does not allow conclusions about advantages or disadvantages about the type of reconstruction and technical details with regard to complications or the functional outcome.

In our case series we confirm that retrocolonic reconstructions are rarely performed and contain individual situations needing adjusted considerations and solutions (e.g. using the Merendino limb as a feeding enterostomy as well as for the distal anastomosis during colonic interposition). However, these operations can be performed safely if surgical expertise in both esophageal and colonic surgery is available.

## Conclusion

In conclusion, secondary and reserve reconstructions with cervical anastomoses, though rare, are feasible with acceptable morbidity. Most patients perceive a discontinuity situation as extremely limiting and are willing to accept a significant perioperative risk to restore continuity. Our cohort confirms the favourable functional outcomes.

### Electronic supplementary material

Below is the link to the electronic supplementary material.

Supplementary Material 1

## Data Availability

No datasets were generated or analysed during the current study.

## Abbreviations

CCS: Charlson-Comorbidity-Score
ASA: American Society of Anaesthesiology
CCI: Comprehensive-Complication-Index
LOS: Length of stay
BMI: Body mass index
COPD: Chronic-obstructive-pulmonary-disease
MIC: Minimally-invasive-surgery
ICU: Intensive-care-unit
IMC: Intermediate-care
FU: Follow-up
QoL: Quality of life
EVT: Endoluminal vacuum therapy
