# Supplementary material for: Surgical options in retrosternal oesophageal reconstruction
Source: Langenbecks Arch Surg. 2024 Aug 3;409(1):238. doi: 10.1007/s00423-024-03433-6 (PMC11297802; doi:10.1007/s00423-024-03433-6)
Supplement: Supplementary file 1 — Supplementary Material 1 [file 423_2024_3433_MOESM1_ESM.docx]

**Supplements:**

Supplement Figure 1a-i: Quality of life scores for all SF 36 items.

Figure 1a: Physical functioning

Legend: QoL: quality of life. Numbers of patients are according to cases shown in Table 1. Colours/Patients: No.1: Blue; No.2: Red. No.3: Green; No.4: violet; No.5: light-blue; No.6: Yellow; No.7: Darkblue; No.8: Brown; No.9: Darkgreen; No.10: Purple; No.11: Petrol; No.12: lightbrown/chestnut.

Figure 1b: Physical limitation

Legend: QoL: quality of life. (4 patients remained at. 0%). Numbers of patients are according to cases shown in Table 1. Colours/Patients: No.1: Blue; No.2: Red. No.3: Green; No.4: violet; No.5: light-blue; No.6: Yellow; No.7: Darkblue; No.8: Brown; No.9: Darkgreen; No.10: Purple; No.11: Petrol; No.12: lightbrown/chestnut.

Figure 1c: Legend: QoL: quality of life. (4 patients remained at. 0%). Numbers of patients are according to cases shown in Table 1. Colours/Patients: No.1: Blue; No.2: Red. No.3: Green; No.4: violet; No.5: light-blue; No.6: Yellow; No.7: Darkblue; No.8: Brown; No.9: Darkgreen; No.10: Purple; No.11: Petrol; No.12: lightbrown/chestnut.

Figure 1d: Energy and fatigue. Legend: QoL: quality of life. Numbers of patients are according to cases shown in Table 1. Colours/Patients: No.1: Blue; No.2: Red. No.3: Green; No.4: violet; No.5: light-blue; No.6: Yellow; No.7: Darkblue; No.8: Brown; No.9: Darkgreen; No.10: Purple; No.11: Petrol; No.12: lightbrown/chestnut.

Figure 1e: Emotional wellbeing. Legend: QoL: quality of life. Numbers of patients are according to cases shown in Table 1. Colours/Patients: No.1: Blue; No.2: Red. No.3: Green; No.4: violet; No.5: light-blue; No.6: Yellow; No.7: Darkblue; No.8: Brown; No.9: Darkgreen; No.10: Purple; No.11: Petrol; No.12: lightbrown/chestnut.

Figure 1f: Social functioning. Legend: QoL: quality of life. Numbers of patients are according to cases shown in Table 1. Colours/Patients: No.1: Blue; No.2: Red. No.3: Green; No.4: violet; No.5: light-blue; No.6: Yellow; No.7: Darkblue; No.8: Brown; No.9: Darkgreen; No.10: Purple; No.11: Petrol; No.12: lightbrown/chestnut.

Figure 1g: Pain.

Legend: QoL: quality of life. Numbers of patients are according to cases shown in Table 1. Colours/Patients: No.1: Blue; No.2: Red. No.3: Green; No.4: violet; No.5: light-blue; No.6: Yellow; No.7: Darkblue; No.8: Brown; No.9: Darkgreen; No.10: Purple; No.11: Petrol; No.12: lightbrown/chestnut.

Figure 1h: Health change.

Legend: QoL: quality of life. Numbers of patients are according to cases shown in Table 1. Colours/Patients: No.1: Blue; No.2: Red. No.3: Green; No.4: violet; No.5: light-blue; No.6: Yellow; No.7: Darkblue; No.8: Brown; No.9: Darkgreen; No.10: Purple; No.11: Petrol; No.12: lightbrown/chestnut.

Figure 1i: General health.

Legend: QoL: quality of life. Numbers of patients are according to cases shown in Table 1. Colours/Patients: No.1: Blue; No.2: Red. No.3: Green; No.4: violet; No.5: light-blue; No.6: Yellow; No.7: Darkblue; No.8: Brown; No.9: Darkgreen; No.10: Purple; No.11: Petrol; No.12: lightbrown/chestnut.
